# HPV vaccines cut cancer cases

**DOI:** 10.1038/s43856-021-00056-6

**Published:** 2021-12-06

**Authors:** Ben Abbott

**Affiliations:** Communications Medicine, https://www.nature.com/commsmed

## Abstract

HPV infection causes the majority of cases of cervical cancer but can be prevented via vaccination. A new study in *The Lancet* reports a substantial reduction in cervical cancer incidence after the introduction of the HPV immunisation programme in England.

**Figure:**
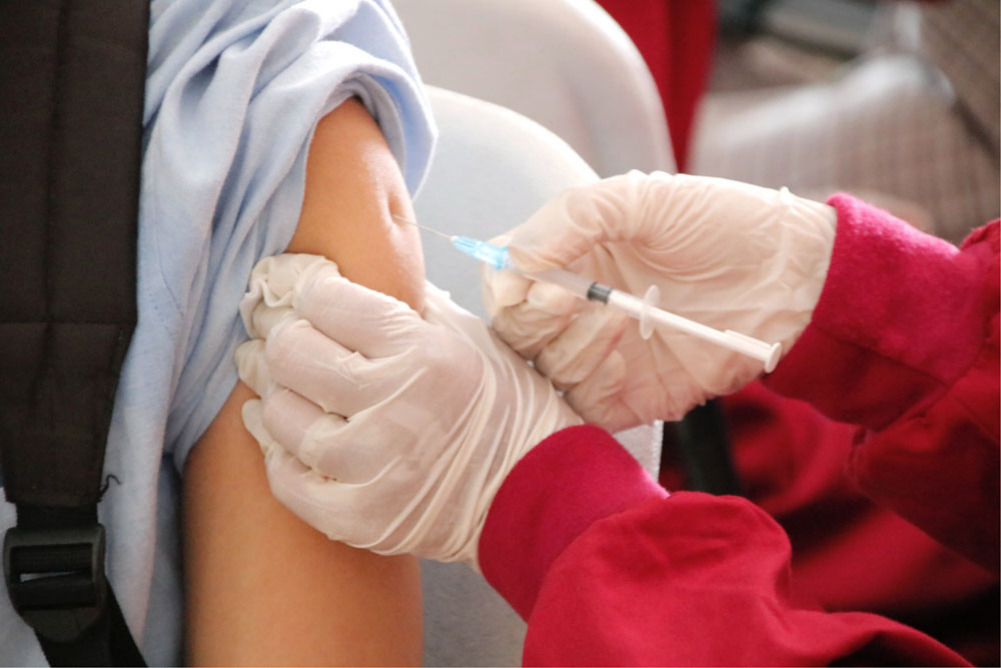
Ed Us on Unsplash

Cervical cancer is one of the most common cancers in women and most cases are caused by human papillomavirus (HPV) infection, particularly high-risk HPV types 16 and 18. HPV vaccination has been shown to protect against HPV infection and cervical intraepithelial neoplasia (CIN)—a precursor to cervical cancer—in clinical trials, but there is little real-world evidence of the effectiveness of HPV vaccines in reducing cancer rates.

The UK began to offer HPV vaccines to school-age girls in 2008, with a bivalent vaccine targeting HPV 16 and 18 called Cervarix. Now, Falcaro and colleagues report population-based cancer registry data to estimate the effect of Cervarix-based HPV vaccination on the incidence of cervical cancer and cervical carcinoma in situ, or grade 3 CIN (CIN3), in women in England^1^.

The authors compare cervical cancer and CIN3 incidence rates in three cohorts who were offered the vaccine at different ages with earlier cohorts who were not offered the vaccine, using several statistical models to account for various confounding factors. They report relative reductions in cancer cases of 34% in those offered the vaccine at age 16–18, 62% for age 14–16, and 87% for age 12–13. Reductions in CIN3 incidence were even greater, reaching 97% in the group aged 12–13.

Although data on vaccine status for individuals within each cohort was not available, these striking results provide strong evidence that HPV vaccination protects against cervical cancer and provide hope that it could be eliminated in the near future. Continued effort will be required to promote vaccine uptake and ensure vaccines reach low- and middle-income countries where access to cervical cancer screening and treatment is limited. Further research is also needed to determine the extent to which vaccination protects against other HPV-associated cancers, such as head and neck cancer, and to determine the impact of the more recently used quadrivalent vaccine Gardasil.
